# Practical Test-Time Domain Adaptation for Industrial Condition Monitoring by Leveraging Normal-Class Data

**DOI:** 10.3390/s25247614

**Published:** 2025-12-15

**Authors:** Payman Goodarzi, Andreas Schütze

**Affiliations:** Laboratory for Measurement Technology, Saarland University, 66123 Saarbrücken, Germany; schuetze@lmt.uni-saarland.de

**Keywords:** domain shift, AutoML, deep learning, condition monitoring, domain adaptation, fault detection, multi-sensor

## Abstract

Machine learning has driven significant advancements across diverse domains. However, models often experience performance degradation when applied to data distributions that differ from those encountered during training, a challenge known as domain shift. This issue is particularly relevant in industrial condition monitoring, where data originate from heterogeneous sensors operating under varying conditions, hardware configurations, or environments. Domain adaptation is a well-known method to address this problem; however, the proposed methods are not directly applicable in real-world condition monitoring scenarios. This study addresses such challenges by introducing a Normal-Class Test-Time Domain Adaptation (NC-TTDA) framework tailored for condition monitoring applications. The proposed framework detects distributional shifts in sensor data and adapts pretrained models to new operating conditions by exploiting readily available normal-class samples, without requiring labeled target data. Furthermore, it integrates seamlessly with automated machine learning (AutoML) workflows to support hyperparameter optimization, model selection, and test-time adaptation within an end-to-end pipeline. Experiments conducted on six publicly available condition monitoring datasets demonstrate that the proposed approach achieves robust generalization under domain shift, yielding average AUROC scores above 99% and low false positive rates across all target domains. This work emphasizes the need for practical solutions to address domain adaptation in condition monitoring and highlights the effectiveness of NC-TTDA for real-world industrial monitoring applications.

## 1. Introduction

Machine learning (ML) has established itself as a valuable tool in daily life, with applications ranging from entertainment and healthcare to industrial automation. ML-based tools demonstrate strong performance across various applications; however, their effectiveness often drops when the data distribution deviates from that of the training set. This phenomenon, known as domain shift or distribution shift, poses a significant challenge to real-world deployments [1]. For example, a model trained to classify handwritten digits using the MNIST [2] dataset (source domain) may perform poorly when tested on street view house numbers (SVHN [3]) dataset (target domain), despite both tasks involving digit recognition. This is because the visual characteristics of the digits (e.g., font, background noise, resolution) vary significantly between the two domains.

The distribution of the target domain may differ from that of the source (training) data. Ideally, models should maintain robustness against such domain shifts. This is especially important in industrial settings, where collecting and labeling new data is costly and in many cases impractical [4,5]. In these cases, the ideal approach is to train a model on one or multiple source domains so that it can generalize to other unseen, but similar domains.

Although numerous methods have been proposed to enhance ML model robustness against domain shift [6,7,8], achieving full invariance to all domain shifts remains challenging [9]. Nevertheless, models can often be made robust to specific types of shifts, such as linear shifts or those confined within a limited range [7]. For instance, in a bearing fault diagnosis system, it may be possible to generalize a model across different rotational speeds within a specific range [10,11]. However, adapting that model to a completely different bearing type or operational condition can be significantly more difficult [12].

Recent advances in addressing domain shift for fault diagnosis have introduced specialized contrastive and graph-based methods to handle challenging industrial scenarios. Chen et al. [13] address surface defect detection on aluminum substrates under dynamic industrial conditions, where traditional CCD/CMOS cameras struggle with minute defects. They propose a Progressive Contrastive Representation Learning framework that combines novel event stream imaging with a four-stage contrastive loss to handle both known and unknown fault classes effectively. Qi et al. [14] propose a multi-task graph isomorphism network enhanced with an attention mechanism that jointly performs fault diagnosis and RUL prediction, leveraging parameter sharing and self-attention to capture common features between different tasks.

Transfer learning in ML refers to the process of applying knowledge gained from one task or domain to another related task or domain. For example, in fault detection, a model trained to detect faults in a milling machine could be adapted to predict the remaining useful life of a different machine operating in another factory. Standard transfer learning scenarios need labeled data from the target domain [15]. When transfer is successful, referred to as positive transfer, the requirement for labeled data is significantly reduced compared to training a model from scratch [16].

Domain adaptation is a specialized form of transfer learning that specifically addresses the problem of domain shift. In a domain adaptation setting, the source and target tasks are identical, but their data distributions differ across domains [6]. For instance, a condition monitoring model trained on data from a gearbox operating under one set of conditions might need to be adapted to a different force or speed level for the same gearbox. Unsupervised domain adaptation (UDA) refers to methods that do not require labeled data from the target domain [17]. UDA methods are particularly useful in industrial applications, where obtaining target domain labels is often difficult or infeasible.

UDA is a common strategy for adapting trained models to new domains [6]. These methods often build on transductive learning principles, leveraging unlabeled data from the target domain to adjust the model specifically for the test set, rather than aiming for generalization to unseen target domains. UDA methods have demonstrated effectiveness across various applications, including classification and regression tasks [18]. A common example of UDA in multi-class classification is training a model on handwritten digits from the MNIST dataset (source domain) and then adapting it to recognize digits in the SVHN dataset (target domain) without relying on labeled data from SVHN. While this example is illustrative, UDA methods have been successfully extended to domains far beyond computer vision [7,8,19].

UDA methods can be classified according to whether source domain training data is accessible during the adaptation process. In conventional UDA, both source data and unlabeled target data are available [18]. However, in source-free domain adaptation (SFDA), only a pre-trained source model is accessible, with no access to the source data itself [20]. SFDA is particularly valuable in privacy-sensitive or data-restricted environments, where sharing raw training data may be legally, ethically, or commercially constrained [21].

Although numerous domain adaptation methods have been effectively applied to condition monitoring, most assume idealized conditions that rarely correspond to real-world industrial settings [15]. For example, assuming continuous access to both source and target data is often impractical because of system limitations, domain privacy, or distribution shifts that evolve over time.

Addressing the practical limitations of conventional domain adaptation methods in condition monitoring, this study proposes a Normal-Class Online Domain Adaptation framework tailored for real-world industrial environments. The key contributions are (1) leveraging automated machine learning (AutoML) for automated optimization across complex multi-sensor scenarios; (2) utilizing only readily available normal-class data for practical and effective test-time adaptation in condition monitoring; (3) generalizing the framework to both deep learning (HP-ConvNet) and conventional FESC methods; (4) comprehensive validation across six real-world condition monitoring datasets.

The remainder of this article is organized as follows: Section 2 reviews domain adaptation methods and their applications in condition monitoring. Section 3 describes the datasets and the proposed method. Section 4 presents the experimental results. Section 5 discusses and analyzes the findings. Finally, Section 6 concludes the paper.

## 2. Related Work

Addressing domain shift has a long history, with foundational methods developed before the deep learning era [16,22]. Traditional approaches are generally classified into instance-based transfer, feature-representation transfer, parameter transfer, and relational-knowledge transfer [16]. These methods mitigate distribution shifts by reweighting source instances [23], learning domain-invariant features [24], sharing model parameters [25], or transferring structured relationships between domains [26].

While these taxonomies remain relevant, recent literature emphasizes categorization based on the availability of source and target domain data and labels, considering the growing significance of data accessibility and privacy in modern applications [27,28]. Table 1 summarizes key distinctions between Transfer Learning (TL), Domain Adaptation (DA), and Domain Generalization (DG) from this perspective.

### 2.1. Domain Generalization

Domain generalization refers to the ideal setting where a model is designed to perform reliably across all types of unseen distribution shifts. In practice, this problem is typically simplified by limiting the scope of the shifts to specific covariates. For instance, in a condition monitoring task within a hydraulic system, the objective can be to develop a model capable of handling variations in valve or cooler conditions robustly [29]. Under the assumption that the domain shift is linear, linear models have been shown to achieve better performance [4].

Nevertheless, no domain generalization method is universally effective across all use cases. Under fair and standardized evaluation protocols, many domain generalization algorithms have been shown to perform no better than standard empirical risk minimization (ERM) [9,30]. This finding challenges the perceived advantages of specialized methods. Furthermore, recent theoretical work underscores a fundamental limitation: to achieve a classifier with excess error no greater than ϵ, any algorithm requires at least a number of distinct training domains that grows polynomially in 1/ϵ, regardless of the amount of data available in each domain [30]. In real-world settings, the number of training domains is often insufficient, which makes achieving reliable generalization to completely unseen distributions challenging.

### 2.2. Domain Adaptation

With the mentioned limitations of domain generalization, domain adaptation emerges as a more practical alternative in real-world scenarios. Domain adaptation is potentially useful when the model is not strictly tied to the source domain and limited access to the target domain is available. Domain adaptation methods can be classified according to the strategies they use to align source and target domains.

#### 2.2.1. Feature Alignment

Feature alignment methods mitigate statistical differences between domains by projecting them into a common feature space [31]. Methods such as Maximum Mean Discrepancy (MMD) [32] and CORrelation ALignment (CORAL) [33] belong to this category.

#### 2.2.2. Adversarial Training

Adversarial training approaches increase domain confusion by introducing a domain discriminator alongside a gradient reversal layer. A prominent example is Domain-Adversarial Neural Networks (DANN) [34], encouraging the feature extractor to produce representations that are indistinguishable across domains.

#### 2.2.3. Hypothesis Transfer

Hypothesis transfer approaches adjust the decision boundaries of classifiers to suit the target domain. This can be achieved through entropy minimization [35], or self-supervised pseudo-labeling [36], which assigns labels to unlabeled target data for iterative refinement.

### 2.3. Source-Free Domain Adaptation

Recently, SFDA has gained increasing attention. These approaches operate under the assumption that source data is inaccessible during the adaptation phase, which is advantageous for preserving data privacy and reducing computational and storage overhead. Representative examples of such approaches include

- Tent [37], which adapts only the batch normalization layers by minimizing prediction entropy on the target data;
- SHOT [20], which freezes the feature extractor and fine-tunes the classifier via pseudo-labeling;
- AdaBN [38], an early method that recalibrates batch normalization statistics using target domain samples.

These methods demonstrate that even without access to source data, it is possible to adapt models effectively.

### 2.4. Test-Time Domain Adaptation

Test-time domain adaptation (TTDA) refers to adapting a pre-trained model using only the target domain data that becomes available during inference time [39]. In contrast to conventional domain adaptation or SFDA, TTDA does not require labeled or unlabeled target data beforehand. Instead, adaptation takes place online, enabling robust deployment in environments where data distributions may evolve or shift unexpectedly, such as in real-world industrial applications with changing operational conditions.

Typical TTDA methods assume either that test samples arrive in batches or that data is available sequentially [40]. Techniques often leverage batch normalization updates, entropy minimization, or self-training to dynamically improve model performance on the target distribution. Tent [37], originally proposed in the context of SFDA, is also one of the earliest and most widely adopted TTDA approaches, as it updates only the batch normalization parameters to minimize output entropy during test time.

### 2.5. Challenges of Domain Adaptation in Industrial Prognostics

DA methods often integrate several loss components, such as task-specific loss, distribution alignment loss, and correlation regularization, each weighted by empirically selected trade-off parameters [33,34,41]. This increases both the computational complexity and the demand for tuning and resources. Furthermore, widely used alignment strategies like MMD, CORAL, and adversarial training frequently suffer from unstable optimization objectives. The shifting alignment targets during training can lead to oscillating losses and slow convergence [7].

While such issues are inherent to domain adaptation, predictive maintenance and condition monitoring introduce additional domain-specific challenges. Although domain adaptation has been widely used to address domain shifts in condition monitoring [15], its application to prognostic tasks remains particularly challenging. This is primarily because, in many real-world scenarios, the target domain data consists only of samples from the normal-class. This condition is frequently observed when a system has recently been deployed or after maintenance procedures have been performed.

## 3. Materials and Methods

This section presents the datasets and methodologies employed in this study.

### 3.1. Datasets

This study uses six publicly available datasets related to industrial condition monitoring. The datasets used in this study are listed below.

- The ZeMA Electromechanical Axis (EA) dataset [42], ZeMA gGmbH, Saarbrücken, Germany;
- The ZeMA Hydraulic System (HS) dataset [29];
- The Open Guided Waves (OGW) dataset [43];
- The Paderborn University Bearing (PU) dataset [44], Paderborn University, Paderborn, Germany;
- The Case Western Reserve University Bearings (CWRU) dataset [45], Case Western Reserve University, Cleveland, OH, USA;
- The Saarland University Bearings (UdS) dataset [46], Saarland University, Saarbrücken, Germany.

Table 2 presents the causes of distribution shifts and the specific operating conditions (OpC) in the source and target domains. For each use case, two distinct target domains are defined. The sources of these domain shifts are varied and may include hardware variations (e.g., the use of different devices), differences in operational settings (e.g., varying motor loads), or system-level changes (e.g., adjustments to cooling capacity). As described in [47], the datasets are balanced or transformed to a balanced version. EA, HS, and CWRU are multiclass classification use cases, while OGW, PU, and UdS are binary classification tasks.

In addition to the datasets utilized in [47], we incorporated the Saarland University Bearings (UdS) dataset [46] into our collection. The UdS dataset contains accelerometer measurements from three cylindrical roller bearings (B10, B20, and B30). This dataset was specifically designed for the systematic analysis of domain adaptation challenges in condition monitoring tasks [46]. Various covariates were intentionally varied in the dataset to serve as potential sources of domain shift. In this study, we focus exclusively on varying the bearing position while keeping all other operating conditions fixed. The baseline condition is defined by bearing B10, a speed range of 383 to 960 rpm, and force levels from 1 to 3. The source domain corresponds to position 2, whereas the target domains are defined by positions 1 and 3, respectively. Further details regarding the datasets and experimental setups are provided in [47].

### 3.2. Methods

We employed two categories of approaches: (1) deep learning methods, and (2) conventional ML methods, specifically a combination of feature extraction, feature selection, and classification, which we refer to as FESC. We begin by outlining each method and then describe the NC-TTDA approach.

#### 3.2.1. HP-ConvNet

Deep learning has achieved notable success across various fields, because of its effectiveness in modeling complex patterns in data. In particular, convolutional neural networks (ConvNets) are well-suited for pattern recognition tasks and have become the standard choice for such applications [48]. Numerous variants of ConvNet architectures have been developed to address different tasks and applications [49].

Despite their effectiveness, two primary challenges are associated with deep learning methods: limited interpretability and difficulty in optimizing the model architecture. Deep neural networks often function as “black boxes,” making it challenging to interpret the reasoning behind their decisions. Addressing this lack of transparency requires dedicated post-hoc analysis or explainable AI techniques [50,51].

In addition, identifying optimal hyperparameters is a non-trivial task, particularly in deep networks with both network architecture and training hyperparameters. Neural Architecture Search (NAS), which automates the selection of architectural hyperparameters in deep neural networks (DNNs), has received considerable attention [52].

In well-studied domains like computer vision and natural language processing, it is often feasible to reuse established architectures across multiple applications [53]. This is largely due to the uniformity in input structure and characteristic feature patterns [54]. By contrast, condition monitoring tasks typically involve heterogeneous sensor inputs, each with distinct characteristics [55]. Depending on the application, the input signals may include vibration, velocity, pressure, current, temperature, or even audio and video data [56,57,58]. In addition, variations in sampling rates across sensors introduce further challenges for data synchronization and fusion. Consequently, the integration and processing of multi-sensor data significantly complicate the task of identifying an appropriate network architecture for a given use case.

**Figure 1:**
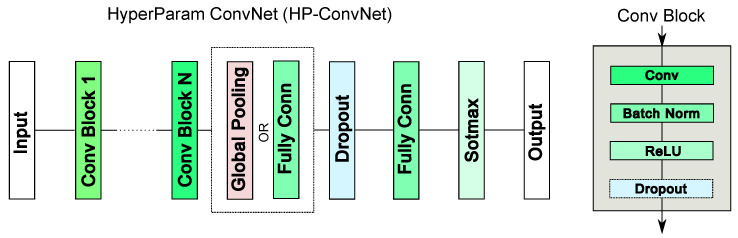
HyperParam ConvNet architecture. The number of convolutional blocks and their properties are defined during hyperparameter optimization [47].

HP-ConvNet [47] addresses these challenges by providing a flexible network architecture specifically designed for hyperparameter tuning in multi-sensor applications. The structure of HP-ConvNet is illustrated in Figure 1, with the search space adopted from [47]. Optimal hyperparameter values are identified through Bayesian optimization [59], which efficiently explores the search space to improve model performance.

#### 3.2.2. FESC

FESC is a well-established approach with demonstrated effectiveness in condition monitoring applications [5,11,55]. It comprises three main components: feature extraction, feature selection, and classification. One key advantage of FESC methods is their inherent interpretability. At each stage, the relationship between extracted features and a model’s predictions remains transparent and can be systematically analyzed. Furthermore, feature extractors can be explicitly designed to produce interpretable sources, enhancing the explainability of the entire process.

However, a key limitation of classical feature engineering is the substantial time and domain expertise required to design effective models. The manual selection and construction of features are often time-consuming and heavily reliant on expert knowledge. Automated machine learning (AutoML) techniques can address this challenge by automating both model selection and hyperparameter optimization [60].

**Figure 2:**
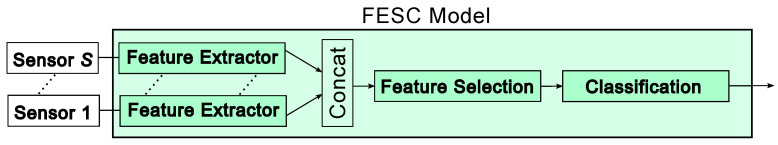
FESC model structure. The model consists of three blocks: feature extraction, feature selection, and classification. Feature extraction is performed separately for each sensor.

Schneider et al. [11] proposed an AutoML framework (Auto-FESC) tailored for condition monitoring, which can automatically identify the optimal FESC configuration for a given task from a predefined search space. In this study, we adopt the Auto-FESC framework from [11]. Figure 2 illustrates the FESC model used in this study, and the corresponding search space is detailed in Table 3. The selected feature extraction methods comprise time domain, time–frequency domain, and frequency domain features to ensure comprehensive coverage of signal characteristics. The feature selection strategies are designed to handle both categorical and non-categorical features. For the final classification stage, we employ linear discriminant analysis with Mahalanobis distance classification (LDAMahal) and support vector machine (SVM) classifiers, enabling the model to capture both linear and nonlinear relationships between features and class labels in various use cases.

#### 3.2.3. HP-Based Deep Ensemble

Hyperparameter optimization is a challenging, yet essential step in machine learning applications, particularly when addressing new tasks or domains. The concept of a hyperparameter-based (HP-based) deep ensemble [47] involves constructing an ensemble model by aggregating multiple models obtained during the hyperparameter optimization process. This ensemble approach offers advantages in producing robust predictions, and the variability among individual model outputs can be leveraged to detect domain and distribution shifts.

The proposed framework is capable of detecting domain shifts [47], and it can be extended to perform domain adaptation based on a hypothesis transfer approach [70]. In practical scenarios in condition monitoring, data collected under healthy or normal operating conditions are typically easily accessible. When transitioning to a new domain, characterized by different working conditions, new data can be gathered to facilitate adaptation of the model. The underlying assumption is that the base models have learned discriminative features that remain informative even in the new domain, although decision boundaries may differ from those in the source domain.

**Figure 3:**
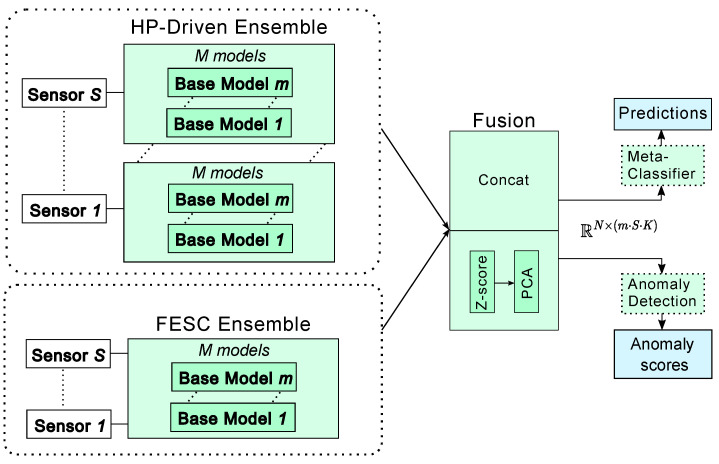
Normal-Class Test-Time Domain Adaptation framework. Either the HP-based deep ensemble or the FESC ensemble can serve as the backbone.

The ensemble model generates a tensor of size $R^{N\times (m\cdot S\cdot K)}$, where *K* is the number of embedded features produced by the base models. The embedded features in the base models can be extracted from different network layers. In general, earlier layers (e.g., Conv Block 1 in Figure 1) generate more generic representations such as simple patterns, while later, deeper layers produce features that are increasingly task-specific and strongly influenced by the training objective [71]. In our framework, features from the last fully connected layer are utilized for prediction and also for anomaly detection. Multi-sensor integration is achieved through independent per-sensor processing by base models, followed by ensemble-level late fusion in a shared feature space. Each base model processes individual sensor data independently, handling sensor-specific changes across domains. Ensemble uncertainty then identifies sensors that become uninformative due to domain shift. Figure 3 illustrates the HP-based deep ensemble as part of the NC-TTDA framework, shown in the top-left box.

To implement this adaptation strategy, a Z-score normalization is first applied to the ensemble features extracted from the target domain data. Subsequently, principal component analysis (PCA) is employed to reduce the dimensionality of each model’s feature set. The number of principal components retained is treated as a hyperparameter; in this study, we used the first and second principal components from each base model’s feature set. This means that the PCA step determines the value of *K* in the tensor used for the anomaly detection output. The concatenated PCA projections from all base models are then combined and used as input for an anomaly detection algorithm to identify deviations from the normal condition. Ensemble diversity creates complementary feature distortions under domain shift, making combined PCA projections more separable from normal-class patterns than single-model features. This effectively converts the initial multiclass classification task into a binary classification problem distinguishing between normal and anomalous states. Following the HP-based deep ensemble method, the *k*-nearest neighbors (*k*-NN) algorithm is employed for anomaly detection. The *k*-NN method classifies a sample based on the distance to its *k* closest neighbors in the feature space, making it a simple yet effective non-parametric approach for detecting abnormal patterns. In this study, the parameter *k* is set to 5, which provides a good balance between sensitivity to local variations and robustness against noise in the data.

The method does not suffer from breaking or forgetting issues during adaptation. The framework applies single-shot test-time adaptation independently for each detected domain shift, avoiding cumulative updates that could lead to forgetting. Each new operating condition uses its own normal-class data to define the anomaly boundary, thereby preserving previously learned information.

For anomaly detection, the additional overhead remains small: first, a Z-score normalization is applied to the extracted features; then, PCA reduces each feature vector to *K* components (typically K=2), and finally, a *k*-NN classifier is applied in this low-dimensional space. The combined PCA and *k*-NN operations introduce only minimal additional cost, on the order of O(m×K), making the overall approach suitable for real-time or near real-time deployment.

#### 3.2.4. FESC Ensemble

The Auto-FESC framework in [11] is based on identifying the optimal combination of FESC methods for each specific task. Beyond selecting the single best model, it is also possible to construct an ensemble method using multiple models obtained from the search algorithm, i.e., the best *m* models.

Figure 3 illustrates the FESC ensemble as part of the NC-TTDA framework, shown in the bottom-left box. Its structure resembles the HP-based deep ensemble; however, the base models differ in that they are derived from FESC methods rather than deep learning models. The input to the meta-classifier is formed by concatenating the ensemble outputs prior to final prediction. Specifically, for SVM classifiers, this corresponds to the distances to the support vectors, while for LDA classifiers, it consists of distances to the class centroids.

The remaining workflow follows a similar approach as in the HP-based deep ensemble, leveraging the combined information of individual models to improve robustness and enable detection of domain shifts.

## 4. Results

In this section, we present the results of NC-TTDA experiments using two ensemble approaches: the HP-driven Deep Ensemble and the FESC Ensemble. We evaluate both methods across the selected benchmark datasets to assess their robustness under domain shift scenarios. Each model is trained on data from a single source domain and evaluated on two unseen target domains. Performance is reported using multiple metrics, including accuracy, area under the receiver operating characteristic curve (AUROC) [72], and false positive rate at 95% true positive rate (FPR95) [73].

**Figure 4:**
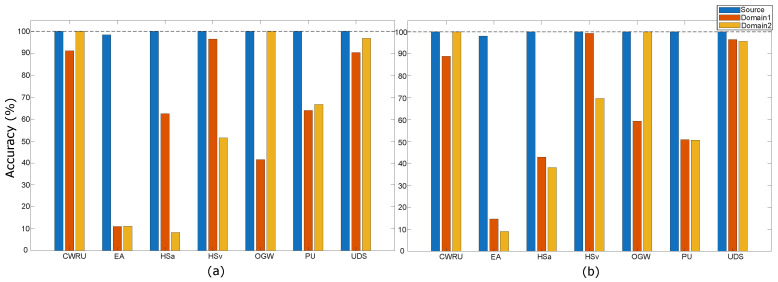
Accuracy of models trained only on the source domain and evaluated on two different target domains before applying NC-TTDA. (**a**) HP-based deep ensemble, and (**b**) FESC ensemble. Each group of bars represents a dataset, with three bars per group corresponding to the source domain, target domain 1, and target domain 2.

Figure 4 presents the classification accuracies of both ensemble methods across all domains before applying NC-TTDA. Each reported value corresponds to the mean over 10 iterations for the respective dataset. The figure illustrates the impact of domain shift on trained ML models, with nearly all datasets showing a substantial drop in prediction accuracy when transitioning from the source to the target domains. Both methods exhibit a similar performance drop under these shifts. Considering that both methods are sophisticated ensemble ML models, this further emphasizes the severity of the problem. Among the datasets, CWRU and UdS experience comparatively small reductions in accuracy, whereas EA and HSa undergo the most pronounced performance degradation.

Table 4 summarizes the AUROC and FPR95 scores for the HP-based deep ensemble and the FESC ensemble after applying NC-TTDA. To perform NC-TTDA, 70% of the target domain data is used as adaptation data, and we sub-sample the abnormal classes to keep the datasets balanced. The reported results represent the average values over 10 iterations on the test set for each dataset. Most models achieve excellent anomaly detection performance on the source domains, often reaching AUROC values of 100.0%, which is desirable and indicates successful training.

**Figure A1:**
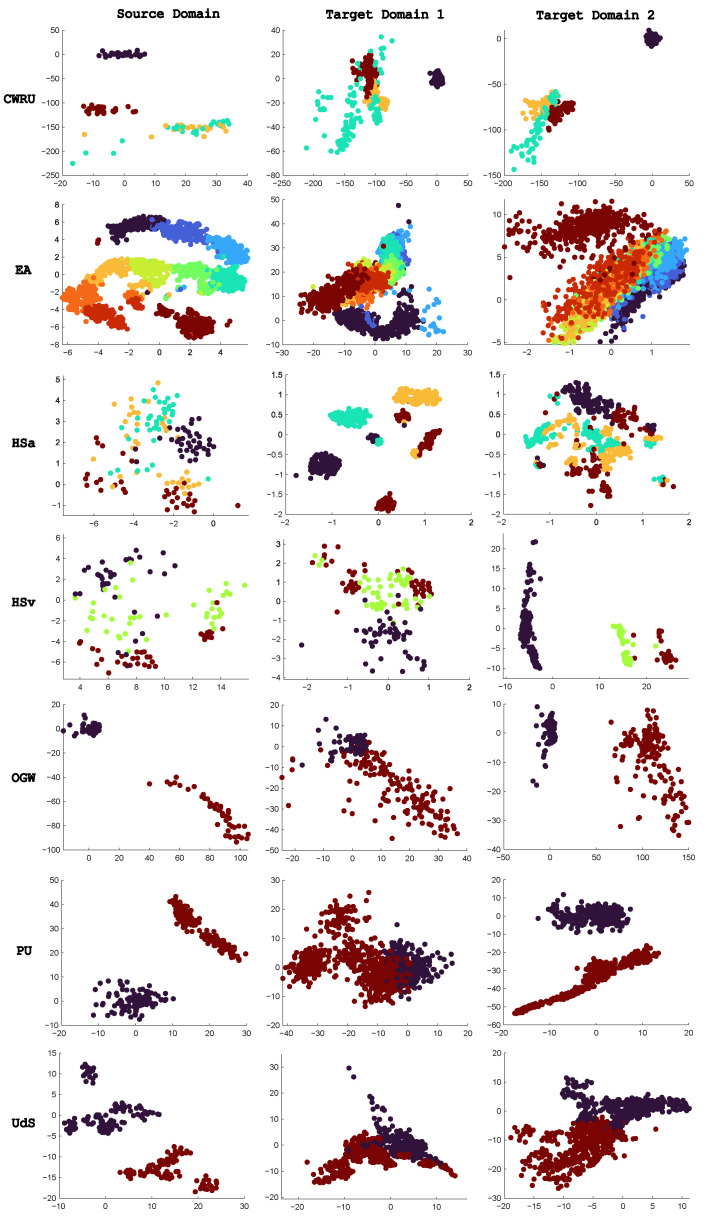
Graphical representation of the embedded features for each dataset and domain produced by the HP-based ensemble models. In all figures, dark blue indicates observations from the normal class, while the other colors represent the remaining classes in the datasets.

**Figure A2:**
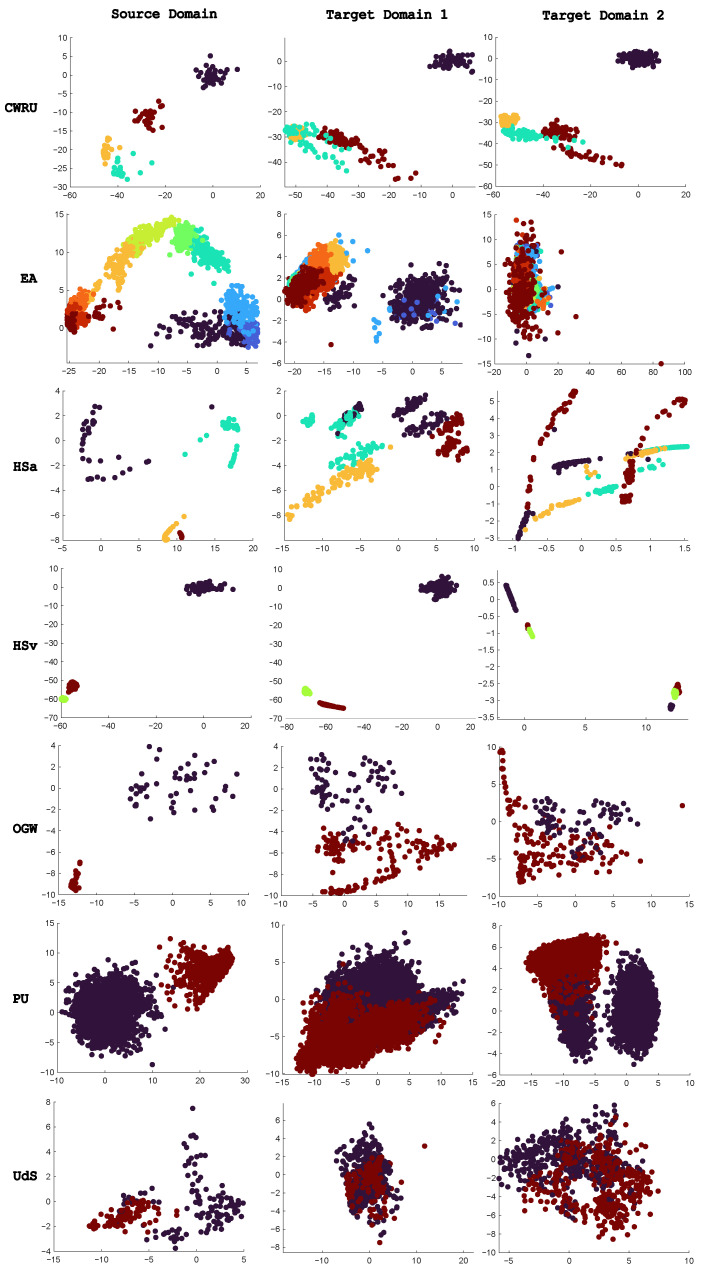
Graphical representation of the embedded features for each dataset and domain produced by the FESC ensemble models. In all figures, dark blue indicates observations from the normal class, while the other colors represent the remaining classes in the datasets.

Applying NC-TTDA on the target domains remains effective even in challenging cases such as EA and HSa. However, some cases exhibit reduced effectiveness. For example, the FESC model performance deteriorates notably for the EA and HS datasets at domain 2, and for the UdS dataset for both target domains. The high FPR in the UdS dataset can be justified by the relatively small drop in target domain accuracy, as illustrated in Figure 4. A minor distribution shift is evident in the UdS use case. The worst-case scenario for the HP-based ensemble occurs in the PU dataset at domain 1, with an AUROC of 94.8% and a relatively high FPR of 18.8%. Visualization of the embedded features helps to interpret the models’ performance. Figure A1 and Figure A2 present PCA plots of the embedded features generated by the HP-based and FESC ensemble models, respectively. The PCA plots show that normal samples from the new domain form distinguishable clusters in the embedding space, despite the substantial differences between the domains.

Table 5 summarizes the average performance metrics of the two NC-TTDA ensemble variants, HP-based and FESC, and compares their results before and after adaptation. The accuracies in Table 5 are the averages of the values reported in Figure 4. On the source domain, both variants achieve near-perfect accuracy, AUROC, and FPR95 scores. In the target domains before applying NC-TTDA, the average classification accuracy drops to 54% for the HP-based method and 5% for the FESC method. Despite this decrease, NC-TTDA maintains strong performance: the HP-based variant reaches an average AUROC of 99% and FPR95 of 3%, while the FESC ensemble attains an AUROC of 96% and FPR95 of 15%.

## 5. Discussion

The consistently high AUROC and low FPR scores on the source domains demonstrate that the proposed NC-TTDA method can be effectively used in combination with the main classification models. Both the FESC and HP-based deep ensemble approaches successfully enable test-time adaptation in previously unseen target domains. However, the varying performance across different datasets and methods underscores the inherent challenges of one-class domain generalization. No direct benchmarks exist for AutoML-based test-time adaptation using only normal-class sensor data. Methods such as Tent, SHOT, and AdaBN require multi-class target data for entropy minimization or pseudo-labeling, making them unsuitable for post-maintenance scenarios where only normal samples are available. Applying these methods in single-class settings typically results in degraded performance. Therefore, our evaluation focuses on baseline comparison, shift detection capability, and one-class domain adaptation effectiveness across six real-world datasets.

Consistent performance drops observed in specific scenarios underscore the significant difficulties posed by domain shifts. Both methods struggle notably with the EA dataset at domain 2, HSa ans HSv at domain 2, and OGW at domain 1. For instance, in the HS dataset, the domain shift arises primarily from differences in cooler performance, quantified as 20% and 3% for target domains 1 and 2, respectively. Intuitively, the severity of domain shift appears greater in domain 2. However, a simple linear correspondence between domains is rarely observed, and the multifaceted nature of domain shift complicates model generalization, necessitating advanced adaptation techniques. Additionally, in the HS datasets, each operating condition includes the primary target fault used for evaluation as well as two additional fault types [29]. Despite this increased complexity, the framework still achieves very high AUROC values and low FPR on both the HSa and HSv datasets after normal-class adaptation.

Both methods exhibit severe difficulties in generalizing to new domains (see Table 5). However, after applying NC-TTDA, the deep ensemble achieves superior average performance. This advantage is from its anomaly detection approach, which leverages feature variance across diverse models to enhance detection capabilities. It is very important that the source domain results after applying NC-TTDA remain intact. This means that as long as the underlying distribution does not change, the models do not generate false positive predictions. Although hyperparameter optimization was conducted for each dataset, the more constrained Auto-FESC search space may have limited its adaptability. While deep ensembles demonstrate superior detection accuracy, FESC methods provide higher interpretability, an important factor in condition monitoring, where explainable model decisions enhance trust and facilitate effective diagnosis.

## 6. Conclusions

This study addresses the critical challenge of domain shift in real-world condition monitoring, where the common domain adaptation assumption of access to labeled target domain data from multiple classes does not hold, particularly in industrial condition monitoring applications. We propose a novel one-class domain adaptation framework that leverages only normal-class data from the target domain at test-time to adapt ensemble models for anomaly detection. This work extend the method from [47], to not only detectiong possible domain shift in test time but also being able to adapt the system in the new domain.

Two ensemble-based approaches were developed and evaluated: the HP-driven deep ensemble and the FESC ensemble. Domain shift scenarios were constructed using six publicly available datasets, and the methods were tested within a single-source domain adaptation setup. Without adaptation, classification accuracy decreases under domain shift due to distributional inconsistency, even when employing sophisticated ensemble methods. By utilizing the proposed NC-TTDA mechanisms, both approaches maintain strong anomaly detection performance across all tested datasets. Experimental results demonstrate that both ensembles achieve high AUROC and low FPR scores on both source and target domains, highlighting their ability to generalize effectively with limited target domain data.

Despite these promising results, some limitations remain. The quantity and quality of unlabeled target domain data critically influence adaptation success. Except for the UdS dataset, the other datasets were not originally designed to explicitly address domain shift, which may affect the generalizability of our conclusions. The approach may fail when the domain shift is so severe that normal and faulty target samples collapse into overlapping regions in the embedding space; in this case domain shifts can still be detected but DA may become unreliable. Furthermore, the current experiments focus solely on single-source domain adaptation, leaving multi-source and continual adaptation as promising directions for enhancing robustness in more complex and heterogeneous industrial environments. Finally, the computational overhead associated with ensemble sizes motivates future work on model compression techniques such as quantization and pruning [74], which could make real-time deployment more feasible.

In this work, two principal components were retained to capture the dominant variance in the ensemble features, reducing noise and computational complexity during adaptation. Although this choice may risk discarding subtle but potentially informative components, the empirical results demonstrate strong anomaly detection performance, indicating that the primary discriminative structure is preserved. Future work could explore adaptive or data-driven component selection strategies to further enhance overall effectiveness.

In summary, this work underscores the practical importance of addressing domain shift in condition monitoring. The proposed AutoML framework not only generalizes well to in-distribution data but also identifies domain shifts and adapts to new distributions in real-world scenarios. Our results demonstrated that integrating NC-TTDA into ensemble frameworks can effectively mitigate performance loss across diverse datasets. These findings highlight the value of combining performance monitoring and anomaly detection with conventional supervised machine learning systems for industrial applications.

## Figures and Tables

**Table 1:** Comparison of Transfer Learning (TL), Domain Adaptation (DA), and Domain Generalization (DG) in terms of data and label availability.

Method	Source Data	Source Labels	Target Data (Train Time)	Target Labels
TL	✓	✓	✓	✓ (few)
DA	✓	✓	✓	–
DG	✓	✓	–	–

“✓” indicates availability or presence, while “–” indicates not available or not applicable.

**Table 2:** Overview of the datasets, including cause of shift and operating condition in source and target domains. Device: different physical actuator instances; OpC: operating conditions.

Dataset	Cause of Shift	Source	Domain 1	Domain 2
EA	Device	Axis 3	Axis 5	Axis 7
HS ^∗^	Cooler	100%	20%	3%
OGW	Pair of sensors	Sensors 1 and 6	Sensors 1 and 2	Sensors 2 and 4
PU	Device group	Group 1	Group 2	Group 3
CWRU	Motor load	1 hp	0 hp	2 hp
UdS	OpC	2	1	3

^∗^ Two classification tasks are generated from the HS dataset, detecting faults of the accumulator (HSa) and valve (HSv), respectively.

**Table 3:** Search space of the Auto-FESC framework [5].

Feature Extraction Methods	
ALA	Adaptive linear approximation [61]
BFC	Best Fourier coefficient [62]
BDW	Best Daubechies wavelets [62]
TFEx	Statistical features in time and frequency domains [55]
NoFE	No feature extraction
PCA	Principal component analysis [63]
StatMom	Statistical moments [11]
Feature Selection Methods	
Pearson	Pearson correlation coefficient [64]
RELIEFF	RELIEFF [65]
RFESVM	Recursive feature elimination
	support vector machines [66]
Spearman	Spearman correlation coefficient [67]
NoFS	No feature selection
Classification Methods	
LDAMahal	Linear discriminant analysis [68] with
	Mahalanobis distance classification
SVM	Support vector machine [69] with
	a radial basis function kernel

**Table 4:** Performance comparison of HP-based deep ensemble and FESC ensemble for seven datasets over three domains after applying NC-TTDA.

(a) HP-Based Deep Ensemble						
	Source		Domain 1		Domain 2	
Datasets	AUROC ↑	FPR95 ↓	AUROC ↑	FPR95 ↓	AUROC ↑	FPR95 ↓
CWRU	100.0	0.0	100.0	0.0	100.0	0.0
EA	98.3	2.2	99.8	0.3	98.3	5.9
HSa	99.2	4.8	99.8	2.4	98.0	9.5
HSv	98.0	5.7	99.4	0.0	99.0	2.4
OGW	100.0	0.0	99.3	5.0	100.0	0.0
PU	100.0	0.0	94.8	18.8	100.0	0.0
UdS	99.8	0.0	99.7	0.1	100.0	0.0
(b) FESC Ensemble						
	Source		Domain 1		Domain 2	
Datasets	AUROC ↑	FPR95 ↓	AUROC ↑	FPR95 ↓	AUROC ↑	FPR95 ↓
CWRU	100.0	0.0	100.0	0.0	100.0	0.0
EA	98.2	5.6	99.6	0.4	86.9	47.3
HSa	100.0	0.0	99.7	0.9	96.4	20.6
HSv	100.0	0.0	100.0	0.0	95.5	29.1
OGW	100.0	0.0	98.6	6.3	100.0	0.0
PU	100.0	0.0	91.9	23.9	99.7	1.4
UdS	100.0	0.0	90.4	52.2	88.9	34.2

↑ indicates that higher values correspond to better performance, while ↓ indicates that lower values correspond to better performance.

**Table 5:** Comparison of the performance between the HP-based and FCSC ensembles. Acc denotes the accuracy of the models before applying NC-TTDA, and AUROC and FPR95 are the results after applying NC-TTDA.

	Source Domain			Target Domain		
Method	ACC	AUROC ↑	FPR95 ↓	ACC	AUROC ↑	FPR95 ↓
HP-based deep ensemble	99.8	99.3	1.8	54.0	99.1	3.2
FESC ensemble	99.7	99.7	0.8	57.0	96.2	15.4

↑ indicates that higher values correspond to better performance, while ↓ indicates that lower values correspond to better performance.

## Data Availability

All datasets used in this study are publicly available. Detailed information on the sources of these datasets can be found in the corresponding sections of this paper.

## Abbreviations

The following abbreviations are used in this manuscript:

AUROC	Area Under the Receiver Operating Characteristic Curve
FPR	False Positive Rate
FPR95	FPR at 95% TPR
kNN	k-Nearest Neighbors
ConvNet	Convolutional Neural Network
HP	Hyperparameter
EA	ZeMA Electromechanical Axis Dataset
HS	ZeMA Hydraulic System Dataset
OGW	The Open Guided Waves Dataset
PU	Paderborn University Bearing Dataset
CWRU	Case Western Reserve University Bearings Dataset
UdS	Saarland University Bearings Dataset
ML	Machine Learning
AutoML	Automated Machine Learning
DNN	Deep Neural Network
NAS	Neural Architecture Search
LDA	Linear Discriminant Analysis
SVM	Support Vector Machines
ALA	Adaptive Linear Approximation
BFC	Best Fourier Coefficient
BDW	Best Daubechies Wavelets
TFEx	Statistical Features in Time and Frequency Domains
NoFE	No Feature Extraction
PCA	Principal Component Analysis
StatMom	Statistical Moments
Pearson	Pearson Correlation Coefficient
RFESVM	Recursive Feature Elimination Support Vector Machines
Spearman	Spearman Correlation Coefficient
NoFS	No Feature Selection
FESC	Feature Extraction Feature Selection, and Classification
DA	Domain Adaptation
UDA	Unsupervised Domain Adaptation
TTDA	Test-time domain adaptation
DANN	Domain-Adversarial Neural Networks
MMD	Maximum Mean Discrepancy
CORAL	Correlation ALignment
OpC	Operating Conditions

## Appendix A

Figure A1 and Figure A2 illustrate the embedded features produced by the HP-based and FESC ensemble models, respectively. Principal Component Analysis (PCA) was employed to reduce the dimensionality of these features for visualization.
